# Fast 3D super-resolution imaging using a digital micromirror device and binary holography

**DOI:** 10.1117/1.JBO.26.11.116502

**Published:** 2021-11-13

**Authors:** Jialong Chen, Zhiqiang Fu, Bingxu Chen, Shih-Chi Chen

**Affiliations:** The Chinese University of Hong Kong, Department of Mechanical and Automation Engineering, Shatin, Hong Kong

**Keywords:** fluorescence imaging, three-dimensional structured illumination microscopy, binary holography

## Abstract

**Significance:** High-speed three-dimensional (3D) super-resolution microscopy is a unique tool to investigate various biological phenomena; yet the technology is not broadly adopted due to its high cost and complex system design.

**Aim:** We present a compact, low-cost, and high-speed 3D structured illumination microscopy (SIM) based on a digital micromirror device and binary holography to visualize fast biological events with super-resolution.

**Approach:** The 3D SIM uses a digital micromirror device to generate three laser foci with individually controllable positions, phases, and amplitudes via binary holography at the back aperture of objective lens to form optimal 3D structured patterns. Fifteen raw images are sequentially recorded and processed by the 3D SIM algorithm to reconstruct a super-resolved image.

**Results:** Super-resolution 3D imaging at a speed of 26.7 frames per second is achieved with a lateral and axial resolution of 155 and 487 nm, which corresponds to a 1.65- and 1.63-times resolution enhancement, respectively, comparing with standard deconvolution microscopy.

**Conclusions:** The 3D SIM realizes fast super-resolution imaging with optimal 3D structured illumination, which may find important applications in biophotonics.

## 1 Introduction

Structured illumination microscopy (SIM) is a fast and effective method to achieve super-resolution imaging and optical cross-sectioning and thus has been widely used in biological studies. In general, a structured light illumination system modulates and shifts the high-frequency contents of a specimen to expand the optical transfer function such that finer features can be resolved by frequency demodulations.^1^ The first three-dimensional (3D) SIM was developed in 2008 that doubles the spatial resolution in all three dimensions.^2^ In this system, a grating is employed to generate 3D structured illumination via three-beam interference, where the light intensity varies both laterally and axially. The phase shifts and rotations of the illumination for frequency demodulations are achieved by mechanical translation and rotations of the grating, which requires careful tuning and has limited imaging speed.

To address the issue, fast 3D SIMs have been developed by replacing the grating with programmable spatial light modulators (SLM) or fast scanning devices;^3^^–^^5^ for example, liquid crystal-based SLMs have been used in 3D SIM to generate high-resolution 3D structured patterns via phase modulations; notably, the temporal resolution is limited by the SLM pattern rate to ∼60  Hz.^3^ To improve speed, a pair of galvanometric scanners and a piezoelectric scanner are combined with a half-wave plate to realize fast control of 3D structured patterns at kilohertz range at the expense of complex system design and higher cost. Compared with these two solutions, DMDs, i.e., a binary SLM, present unique advantages in 3D SIM in terms of speed (up to 32.5 kHz), cost, and compact system design.^5^ The use of DMDs can be divided into projection and holography modes. In the projection mode, the desired grating pattern is directly displayed on the DMD to generate a structured pattern. However, in this system, the diffracted zeroth-order beam has much higher intensity than the ±1st beams, resulting in low-contrast structured patterns that are difficult to reconstruct high-frequency features.^5^ In the holography mode, designed binary holograms are displayed on the DMD to manipulate the wavefront of the incident laser to perform fast beam shaping and random-access scanning.^6^^–^^9^ In comparison to the projection mode, the holography mode offers improved flexibility and precision in generating and controlling the position, phase, and intensity of multiple foci at the back focal plane of an objective lens, which may be used to design and create complex two-dimensional (2D) and 3D structured patterns with improved contrast.

In this work, we present a 3D SIM based on binary holography to realize super-resolution imaging on a wide-field microscope, which achieves a lateral and axial resolution of 155 and 487 nm, respectively, and an imaging speed of 26.7 frames/s (limited by the camera). In this system, a DMD is used to generate 3D structured patterns by the interference of three laser foci with designed positions, phases, and intensities encoded in the hologram. The new 3D SIM system presents improved flexibility in adjusting and fine-tuning the geometry, periods, orientations, and contrast of the 3D structured pattern, leading to a compact DMD-based 3D SIM system with improved imaging speed and signal-to-noise ratio (SNR).

## 2 System Design

### 2.1 Optical Configuration of the Holography-Based 3D SIM

**Fig. 1:**
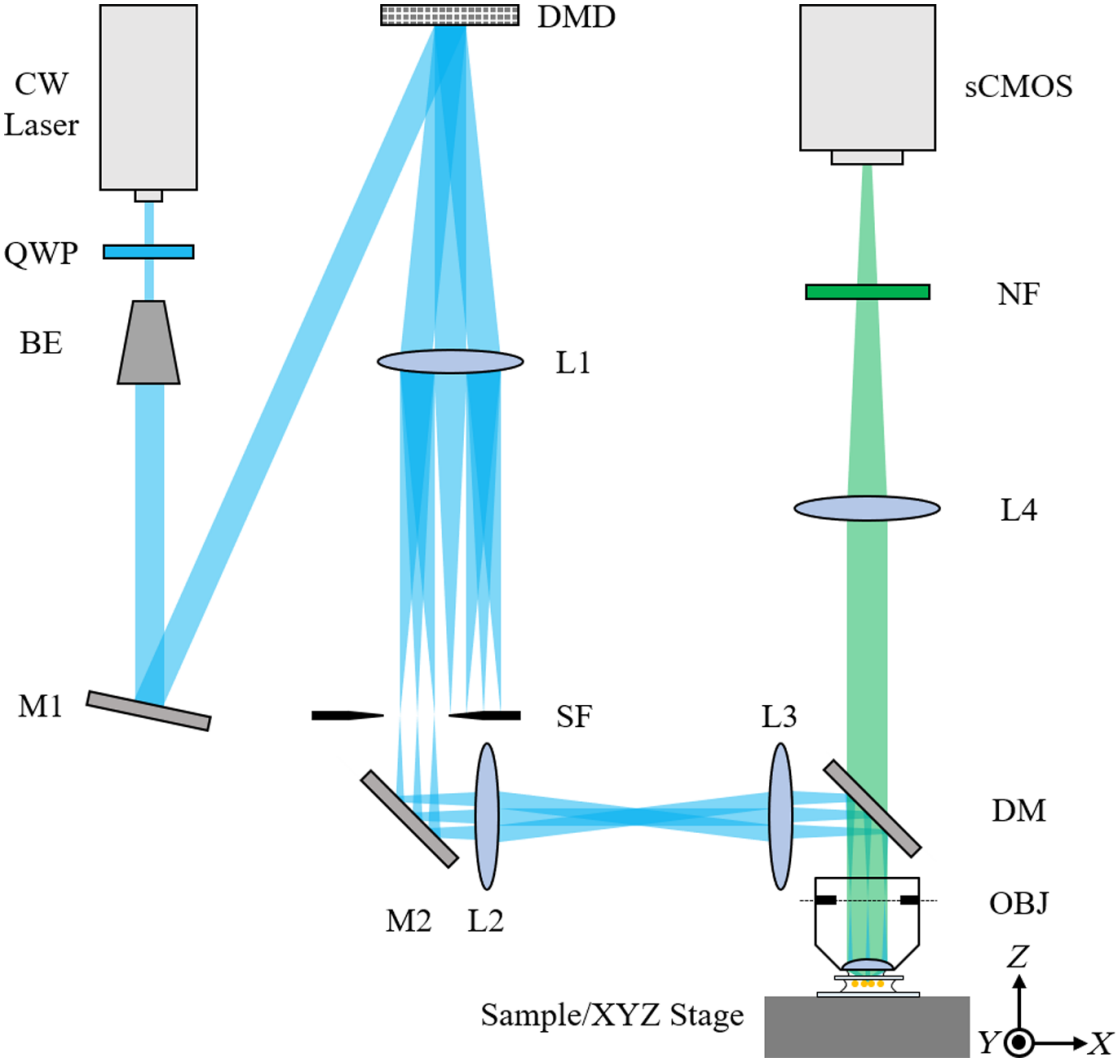
Optical configuration of the holography-based 3D SIM. QWP, quarter-wave plate; BE, beam expander; M1-M2, high reflectivity mirrors; L1–L4, lenses ($f_{L1}=400\;\mathrm{mm}$, $f_{L2}=f_{L3}=200\;\mathrm{mm}$, $f_{L4}=400\;\mathrm{mm}$); SF, spatial filter; DM, dichroic mirror; OBJ, objective lens; NF, notch filter; and sCMOS, scientific complementary metal-oxide semiconductor camera.

Figure 1 shows the optical configuration of the holography-based 3D SIM system. The laser source is a 488-nm continuous-wave laser (MLD, Cobolt). First, a quarter-wave plate (WPQ10M-488, Thorlabs) is used to generate a circularly polarized beam, which ensures the same modulation depth can be achieved for structured patterns of different orientations.^10^ Next, the laser beam passes through a beam expander and a high reflectivity mirror (M1) to fully fill the aperture of a DMD (DLP 4500, Texas Instrument) with uniform illumination. To optimize the power efficiency, the laser incident angle is set to 21.42 deg in reference to the DMD normal to meet the blazing condition.^6^^,^^7^ As the designed wavefront information is encoded in the non-zeroth-order diffractions, a spatial filter is employed to spatially select the -1st-order diffraction beam. Next, the selected diffraction beam is relayed to the back focal plane of the objective lens (OBJ, UPLSAPO 60XW, NA = 1.2, Olympus), thereby forming the designed 3D structured pattern via a three-beam interference at the front focal plane. For detection, the emissions from the specimens are first collected by the objective lens, then pass through a dichroic mirror (DM, T495lpxr, Chroma), tube lens (L4), notch filter (NF, NF488-15, Thorlabs), and lastly recorded by a scientific complementary metal–oxide–semiconductor (sCMOS, C13440, Hamamatsu) camera. A precision XYZ stage (LPS-45, Physik Instrumente) is used to maneuver the specimen for 3D imaging.

### 2.2 Generation of Optimal 3D Structured Patterns

In this section, we present the generation and optimization of three laser foci based on binary holography for forming the 3D structured patterns, where the weighted Gerchberg–Saxton (WGS)^11^ algorithm is applied to improve the quality of the laser foci. To begin, the lateral position of a single laser focus can be mathematically described and controlled by a phase φ(x,y) equation, i.e., tilted wavefront, as expressed as

$$\varphi (x,y)=\frac{2\pi }{\lambda f_{L1}}[x(x_{b}+\Delta x)+y(y_{b}+\Delta y)]+\theta ,$$ (1)

where λ is the excitation wavelength; $x_{b}$ and $y_{b}$ are the applied biased positions in the x and y directions of the −1st diffraction beam for separating the different diffraction orders; Δx and Δy are the applied positions of the laser focus at the back focal plane; and θ is a term to adjust the phase of the focus. To generate and control multiple laser foci, the required phase $\varphi^{\prime }(x,y)$ can be calculated by superposing the single-focus complex phases.^11^ Next, the WGS algorithm is applied to each designed complex phases, where a weighting factor, $w_{n}$, is introduced to optimize the accuracy of the laser focus intensities, as expressed in Eq. (2):

$$\varphi^{\prime }(x,y)=\arg[\underset{n}{\sum }w_{n}e^{j\varphi_{n}(x,y)}],$$ (2)

where $\varphi_{n}(x,y)$ is the phase of the n’th focus obtained from Eq. (1). To obtain the designed intensity ratio, the optimal $w_{n}$ is iteratively updated with respect to the complex amplitudes $V_{n}$ of each focus through Eqs. (25):

$$H(i,j)=\{\begin{array}{ll} 1, & -\frac{q}{2}\leq \frac{\varphi^{\prime }(x,y)}{2\pi }+k\leq \frac{q}{2}\\ 0, & \text{otherwise} \end{array},$$ (3)

$$V_{n}=\overset{I}{\underset{i=1}{\sum }}\overset{J}{\underset{j=1}{\sum }}H(i,j)e^{-j\varphi_{n}(x,y)},$$ (4)

$$w_{n,s}=w_{n,s-1}\frac{\left\langle |V_{n}/V_{t,n}|\right\rangle }{\left|V_{n}/V_{t,n}\right|},$$ (5)

where H(i,j) (1≤i≤I; 1≤j≤J) is the binary hologram generated based on $\varphi^{\prime }(x,y)$ and Lee holography;^12^ 1 and 0 refer to “on” and “off” states of the DMD pixel; I and J are the size of DMD chip; x=i p; y=j·p; p is the DMD pixel size; k is an integer; q is a constant that determines the duty cycle of binary patterns; $w_{n,s}$ is the updated weighting of the n’th focus in the s’th iteration; $V_{t,n}$ represents the target (normalized) amplitude of the n’th focus; and ⟨·⟩ denotes the average operation. To begin, we first assign the target amplitude in Eq. (2), i.e., the initial value of $w_{n,0}$ is set based on $V_{t,n}$. After 5 to 10 iterations, the optimal $w_{n}$ can be identified to improve the accuracy of the amplitude $\left|V_{n}/V_{t,n}\right|$ among the three laser foci with a converging condition that max$||V_{n}/V_{t,n}|-1|<0.01$. Last, the calculated $w_{n}$ are substituted into Eq. (3) to generate the optimal binary hologram for imaging experiments.

## 3 Experiments

**Fig. 2:**
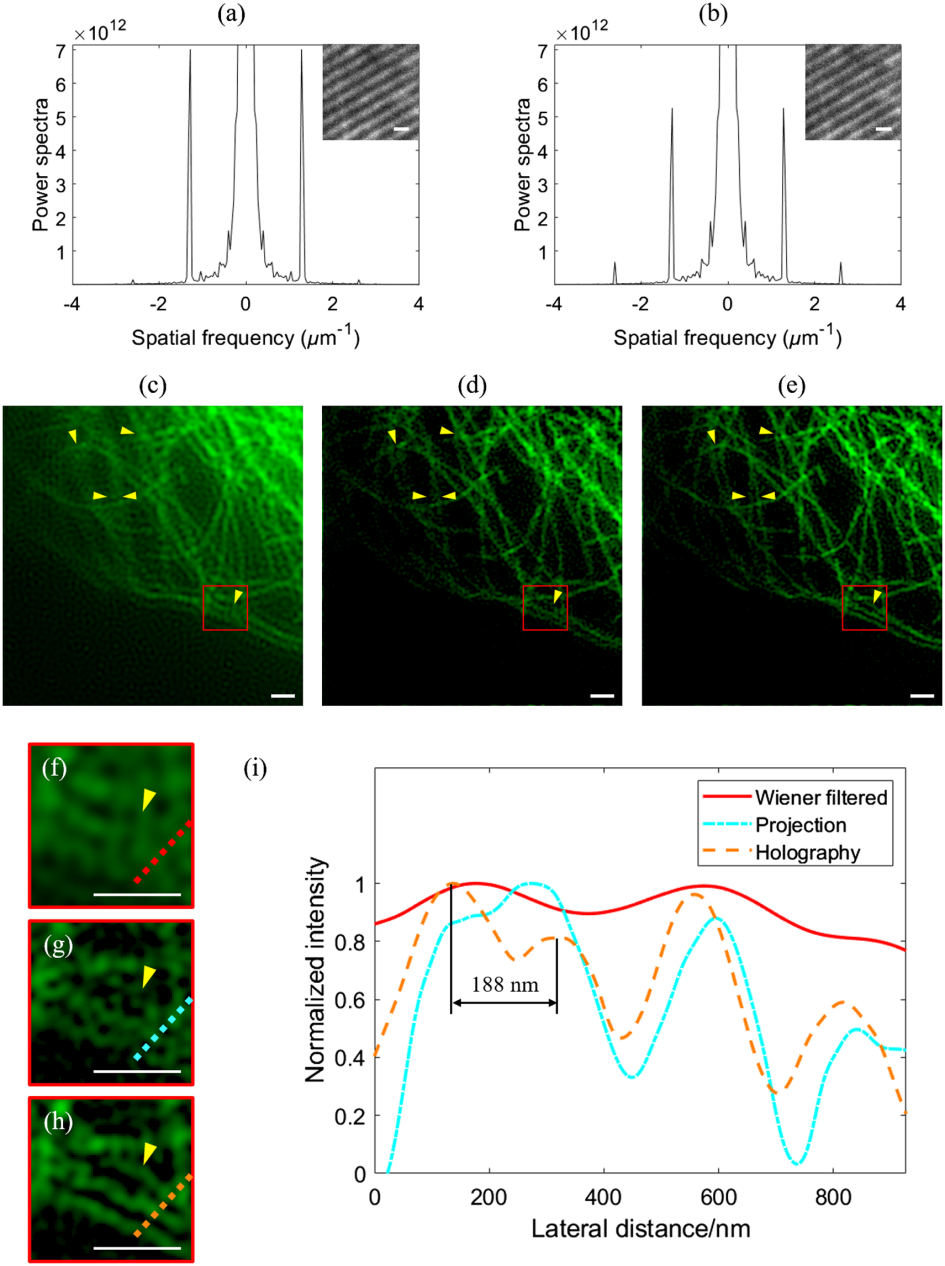
Comparison between projection-based and holography-based DMD 3D SIM. (a), (b) Power spectra and the in-focus emissions of the generated structured patterns via projection-based and holography-based methods, respectively. (c)–(e) Imaging results of the microtubules in U2OS cells (stained with Alexa Fluor 488) reconstructed by the Wiener filter algorithm, projection-based, and holography-based methods, respectively. (f)–(h) Enlarged views of the red boxes in (c)–(e), respectively. (i) Intensity profiles of the color-labeled dashed lines in (f)–(h), respectively. Scale bar: 1  μm.

We first perform imaging experiments to demonstrate the advantages of holography-based DMD 3D SIM over projection-based systems. Figures 2(a) and 2(b) show the measured power spectra and in-focus emissions of the structured patterns with the same modulated second-order frequency at $2.6\;{\mu m}^{-1}$, generated by the projection-based and holography-based DMD 3D SIM, respectively. In the projection mode, the amplitude ratio between the center focus (i.e., zeroth-order beam) and outer focus (i.e., ±1st-order beam) is π/2 according to the grating theory; in the holography mode, the amplitude ratio can be arbitrarily set; and an optimal value of 0.7 is used based on Ref. 2. From the results in Figs. 2(a) and 2(b), one may find that the holography-based SIM produces a four times stronger second-order modulation of the structured pattern in comparison to the projection-based SIM. This ensures good reconstruction of high-frequency features in 3D SIM and improved robustness against noises in high-speed imaging applications. Figures 2(c)–2(e) show 3D imaging experiments on microtubules (U2OS cells stained with Alexa Fluor 488) at an image acquisition rate of 400 frames/s (i.e., 26.7 frames/s after image reconstruction) via the Wiener filter algorithm, projection-based and holography-based approaches, respectively. In the experiment, 15 raw images illuminated with different structured patterns (i.e., five equally spaced phase shifts and each with three equally spaced orientations) are sequentially recorded and processed by an open-source 3D SIM algorithm, i.e., fairSIM in ImageJ.^13^ To better compare the imaging results, Figs. 2(f)–2(h) show zoom-in views of the red boxes in Figs. 2(c)–2(e), respectively; and Fig. 2(i) shows the intensity profiles along the color-labeled dashed lines in Figs. 2(f)–2(h). From the results, one may find that the holography-based SIM, shown in Figs. 2(e), 2(h), and 2(i), generates super-resolved images with higher quality and reduced artifacts; this also shows the importance of second-order modulation for 3D SIM reconstruction.

**Fig. 3:**
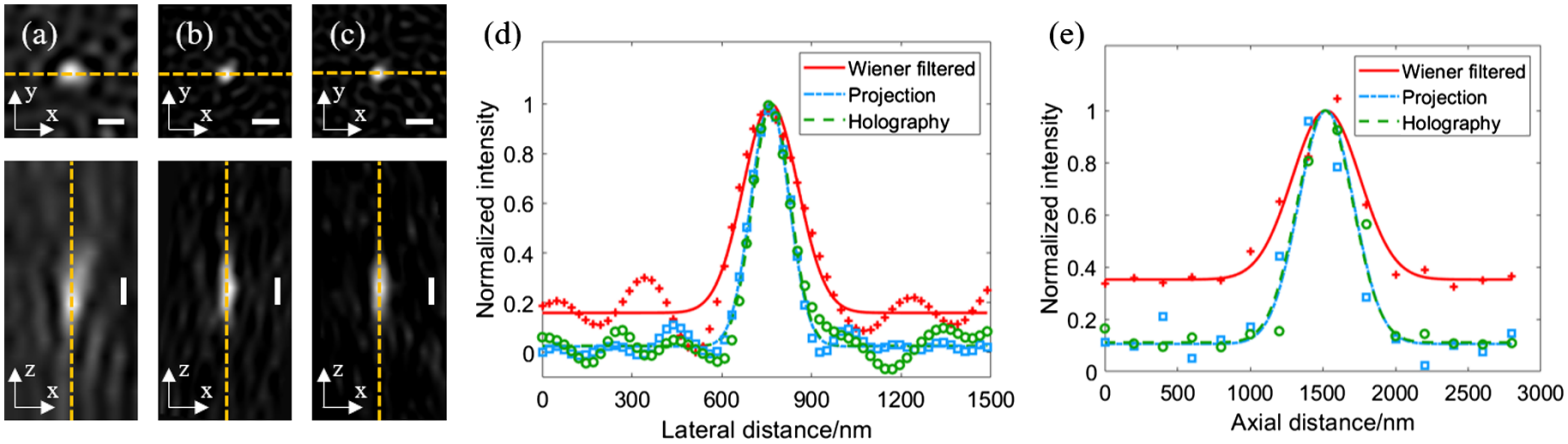
Imaging results of 100-nm-diameter fluorescent microspheres: XY and XZ cross-sectional views of 100 nm microspheres based on the (a) Wiener filter, (b) projection-based 3D SIM, and (c) holography-based 3D SIM; (d) lateral and (e) axial intensity profiles of the dashed lines in (a)–(c). Scale bar: 300 nm.

To characterize the resolution of the holography-based 3D SIM, we perform imaging experiments on fluorescent microspheres (Ø=100  nm, F8803, ThermoFisher), where the raw images are acquired at 100 frames/s (i.e., 6.7 frames/s after reconstruction). In the experiment, 15 raw images with different structured patterns and a modulated second-order frequency of $2.6\;{\mu m}^{-1}$ are used to reconstruct a super-resolved image. Figures 3(a)–3(c) show the imaging results in the XY and XZ planes based on the Wiener filter algorithm, projection-based and holography-based 3D SIM, respectively. Figures 3(d) and 3(e) show the lateral and axial intensity profiles across the dashed lines in Figs. 3(a) and 3(b), respectively. To quantify the resolution, we measured the full-width at half-maximum of intensity profiles in Figs. 3(d) and 3(e); the results indicate that the holography-based 3D SIM achieves a lateral and axial resolution of 155 and 487 nm, respectively; the projection-based 3D SIM achieves a lateral and axial resolution of 159 and 482 nm, respectively; and the conventional Wiener filter algorithm achieves a lateral and axial resolution of 256 and 796 nm, respectively. Note that it is expected that projection-based and holography-based 3D SIM have similar resolution under proper illumination; and the holography-based system will have more advantage in low-light or high-speed imaging conditions due to enhanced second-order modulation, which has been demonstrated in Fig. 2. Comparing with the Wiener filter method (i.e., standard deconvolution microscopy), the holography-based 3D SIM has effectively improved the lateral and axial resolution by a factor of 1.65 and 1.63, respectively.

**Fig. 4:**
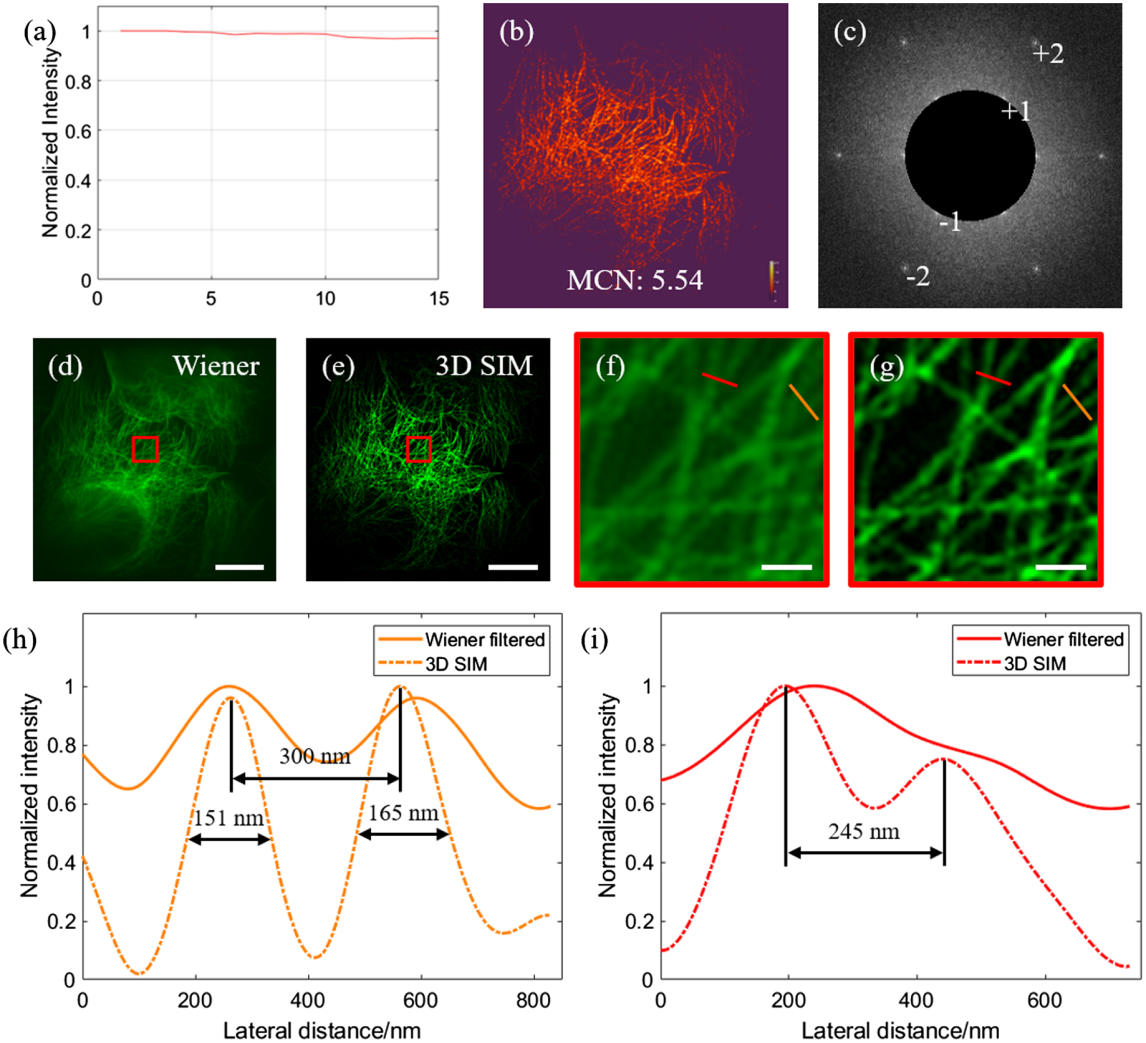
Imaging results of microtubules: (a)–(c) SIMcheck output of channel intensity profile; MCN ratio; and raw Fourier projection, respectively; (d) and (e) reconstructed images based on the Wiener filter and 3D SIM, respectively; (f) and (g) zoom-in views of the boxed regions in (d) and (e), respectively; (h) and (i) intensity profiles of the orange and red lines in (f) and (g), respectively. Scale bars: 10  μm in (d) and (e) and 1  μm in (f) and (g).

Next, we perform imaging experiments on biological specimens, i.e., microtubules in U2OS cells stained with Alexa Fluor 488, to verify the resolution. Before image reconstruction, the collected raw images are first diagnosed by the SIMcheck^14^ toolbox in ImageJ, which is a common practice for assessing the quality of the raw data for SIM. Figures 4(a)–4(c) show three SIMcheck results including the channel intensity profile, modulation contrast-to-noise ratio, and raw Fourier projection. The channel intensity profile compares the average intensity in different recorded frames, and a total intensity variation of 3.16% indicates high excitation stability and low photobleaching. The MCN compares the pattern strength and noise strength, which are related to the quality and reliability of the reconstructed super-resolved features; an average MCN value of 5.54 suggests our raw data are adequate for SIM reconstruction. The raw Fourier projection calculates the maximum intensity projection of the Fourier transform of raw data. In Fig. 4(c), the ±1st and ±2nd orders are clearly visible and clean, indicating good robustness of our system. Figures 4(d) and 4(e) show the reconstructed images based on the Wiener filter algorithm and 3D SIM, respectively. Figures 4(f) and 4(g) show zoom-in views of the red boxes in Figs. 4(d) and 4(e), respectively. From the results, one can clearly observe that the background emissions are better suppressed in the 3D SIM system. Last, Figs. 4(h) and 4(i) show the intensity profiles across the orange and red lines in Figs. 4(f) and 4(g), respectively. The results confirm that the holography-based 3D SIM has effectively improved the imaging resolution to resolve fine features with sizes down to ∼150  nm. This is consistent with the resolution characterization results shown in Fig. 3.

**Fig. 5:**
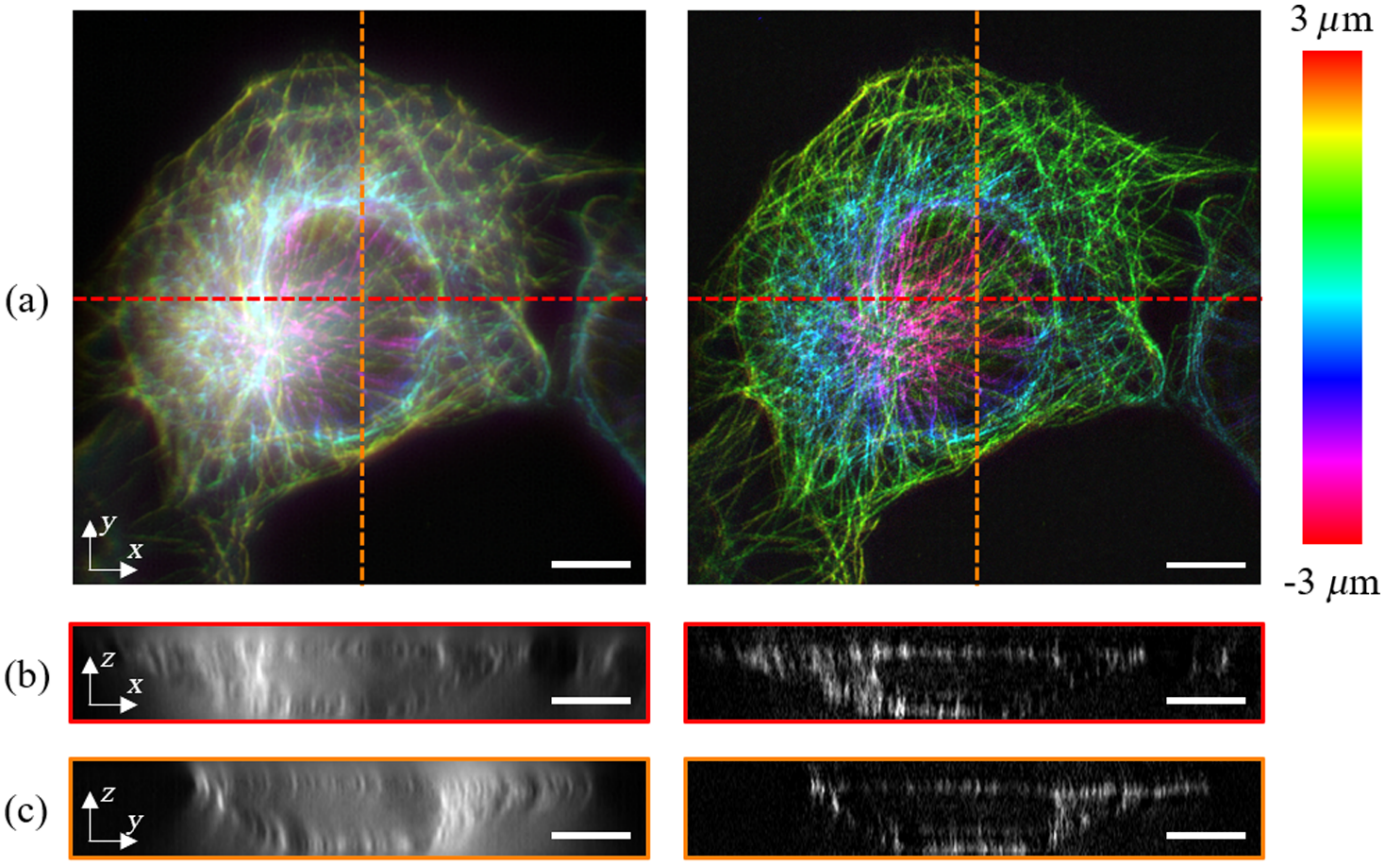
3D imaging of microtubules specimen stained with Alexa Fluor 488: (a) reconstructed 3D images based on the Wiener filter (left) and 3D SIM (right), where the color bar indicates depth; (b) XZ and (c) YZ cross-sectional views along the red and orange dashed lines in (a). Scale bar=5  μm.

Last, 3D imaging is performed on the microtubules specimen. The raw data consist of 30 optical cross-sections, where the distance between adjacent sections is 200 nm. Figure 5(a) shows the reconstructed 3D image in color-coded 2D maps based on the Wiener filter algorithm (left) and 3D SIM (right), where different colors represent optical sections obtained at different depths. From the XZ and YZ cross-sectional views shown in Figs. 5(b) and 5(c), one can observe the substantial resolution enhancement in both lateral and axial directions.

For efficiency, the overall power efficiency is measured to be ∼1% (i.e., 100 mW for the input and 1 mW for the output beam after the objective lens), where most energy is lost at the DMD due to diffraction.^7^ For imaging applications, the illumination power can be easily compensated by increasing the laser power. Note that all imaging results in Figs. 2–5 were obtained at a power level of 0.2 to 0.5 mW, measured at the focal region.

## 4 Conclusion

We have presented a new 3D SIM achieving a lateral and axial resolution of 155 and 487 nm, respectively, and an imaging speed of 26.7 frames/s. In the system, a DMD is used to generate 3D structured illumination based on binary holography. To generate the optimal structure patterns (and thereby the SNR and imaging resolution), the WGS algorithm has been applied to improve the accuracy of the intensity ratio among the three laser foci at the back focal plane of the objective lens. 2D and 3D imaging experiments on fluorescent microspheres and microtubules have been carefully devised and performed on both the holography-based 3D SIM and deconvolution microscopy to measure the 3D point-spread function as well as to demonstrate the resolution enhancement in both lateral and axial directions. These promising results suggest our new 3D SIM platform, which is simple and compact, may find important applications in field of biophotonics.
